# General anesthesia but not conscious sedation improves functional outcome in patients receiving endovascular thrombectomy for acute ischemic stroke: A meta-analysis of randomized clinical trials and trial sequence analysis

**DOI:** 10.3389/fneur.2022.1017098

**Published:** 2022-09-14

**Authors:** Chia-Wei Lee, Yang-Pei Chang, Yen-Ta Huang, Chung-Hsi Hsing, Yu-Li Pang, Min-Hsiang Chuang, Su-Zhen Wu, Cheuk-Kwan Sun, Kuo-Chuan Hung

**Affiliations:** ^1^Department of Neurology, Chi Mei Medical Center, Tainan City, Taiwan; ^2^Department of Neurology, Kaohsiung Municipal Ta-Tung Hospital, Kaohsiung Medical University, Kaohsiung City, Taiwan; ^3^Department of Neurology, Kaohsiung Medical University Hospital, Kaohsiung Medical University, Kaohsiung City, Taiwan; ^4^Department of Surgery, College of Medicine, National Cheng Kung University Hospital, National Cheng Kung University, Tainan City, Taiwan; ^5^Department of Anesthesiology, Chi Mei Medical Center, Tainan City, Taiwan; ^6^Department of Medical Research, Chi Mei Medical Center, Tainan City, Taiwan; ^7^Department of Internal Medicine, Chi Mei Medical Center, Tainan City, Taiwan; ^8^Department of Emergency Medicine, E-Da Hospital, Kaohsiung City, Taiwan; ^9^College of Medicine, I-Shou University, Kaohsiung City, Taiwan

**Keywords:** stroke, endovascular thrombectomy, general anesthesia, sedation, prognosis

## Abstract

**Background:**

This study aimed at comparing the difference in prognostic outcomes between patients receiving general anesthesia (GA) and conscious sedation (CS) for endovascular thrombectomy after acute ischemic stroke.

**Methods:**

Databases from Medline, Embase, Google scholar, and Cochrane library were searched for randomized controlled studies (RCTs) comparing patients undergoing GA and CS for endovascular thrombectomy following anterior circulation ischemic stroke. The primary outcome was frequency of 90-day good functional outcome [defined as modified Rankin Scale score of ≤ 2], while secondary outcomes included successful recanalization rate (SRR) [i.e., modified thrombolysis in cerebral infarction = 2b or 3], mortality risk, symptomatic intracranial hemorrhage (ICH), procedure-related complications, hypotension, pneumonia, neurological outcome at post-procedure 24–48 h, and puncture-to-recanalization time.

**Results:**

Six RCTs including 883 patients published between 2016 and 2022 were included. Merged results revealed a higher SRR [risk ratio (RR) = 1.11, 95% CI: 1.03–1.2, *p* = 0.007; *I*^2^ = 29%] and favorable neurological outcomes at 3-months (RR = 1.2, 95% CI: 1.01–1.41, *p* = 0.04; *I*^2^ = 8%) in the GA group compared to CS group, without difference in the risk of mortality (RR = 0.88), symptomatic ICH (RR = 0.91), procedure-related complications (RR = 1.05), and pneumonia (RR = 1.9) as well as post-procedure neurological outcome (MD = −0.21) and successful recanalization time (MD = 3.33 min). However, GA was associated with a higher risk of hypotension compared with that of CS.

**Conclusion:**

Patients with acute anterior circulation ischemic stroke receiving GA were associated with a higher successful recanalization rate as well as a better 3-month neurological outcome compared to the use of CS. Further investigations are warranted to verify our findings.

**Systematic review registration:**

www.crd.york.ac.uk/prospero/display_record.php?ID=CRD42022342483, identifier: CRD42022342483.

## Introduction

Endovascular thrombectomy (EVT) has revolutionized the treatment of acute ischemic stroke (AIS) with large-vessel occlusion in the anterior circulation since 2015 when several clinical trials demonstrated its efficacy for reperfusion (1, 2). Several prognostic factors have been identified for the achievement of better neurologic outcome of EVT, including a shorter reperfusion time and a stable hemodynamic condition during the procedure (3–5). Consistently, one observational study reported a 10% reduction in the probability of a good outcome for every 15-min delay in EVT reperfusion (6), highlighting the importance of shortening the door-to-reperfusion time. For patients receiving EVT, the most common anesthetic modalities include general anesthesia (GA) and conscious sedation (CS), both of which have their pros and cons (7–9). The choice of the optimal anesthetic approach to EVT is still under debate. Observational studies comparing GA with other strategies (i.e., local anesthesia or CS) have reported poorer outcomes in patients receiving GA for EVT (10–12). In contrast, pooled evidence from a recent meta-analysis (7) focusing on five randomized controlled trials (RCTs) (13–18) demonstrated favorable successful recanalization rate (SRR) and functional outcomes associated with GA compared to CS. Nevertheless, the limited sample size in that meta-analysis (i.e., 498 patients) (7) may impair the robustness of their findings. Recently, one multicenter RCT involving 351 patients from France showed comparable functional outcomes between patients receiving GA and those undergoing CS for EVT (19). Taking into account the limitations of the previous meta-analysis (7) and the availability of updated data, we conducted this systematic review and meta-analysis to provide more evidence for clinical decision.

## Methods

This review was reported according to the Preferred Reporting Items for Systematic Reviews and Meta-Analyses (PRISMA) statement guidelines and was registered at the PROSPERO international database (CRD42022342483).

### Search strategy and studies selection

We searched the databases of Embase, Medline, and the Cochrane controlled trials register for peer-reviewed RCTs comparing the prognostic outcomes between GA with CS in patients requiring EVT using the keywords “general anesthesia,” “conscious sedation,” “stroke” or “thrombectomy” and their synonyms as well as controlled vocabulary from inception to June 28, 2022. There were no restrictions on age, language, gender, publication date, sample size, and geographic location during literature search. We also reviewed relevant meta-analyses to retrieve related articles. The syntax and search strategies for one of these databases (i.e., Medline) is illustrated in Supplementary Table 1.

After removal of duplicated citations, two independent authors examined the titles and abstracts of the remaining records to determine the eligibility for inclusion before a full-text review. Disagreements between the two authors were settled by consensus or discussion with a third author.

### Study selection criteria

Studies were considered to be eligible for inclusion if the following criteria were fulfilled: (a) Population: adult patients (i.e., ≥18 years) receiving EVT for acute anterior circulation ischemic stroke regardless of timing of symptom onset (i.e., <6 h or ≥6 h), (b) Intervention: use of GA as an anesthetic approach (GA group) regardless of the thrombectomy technique, (c) Comparison: CS with or without the use of local anesthetics (CS group). CS was defined as the use of sedative or/and analgesic agents via intravenous route to provide sedative, amnesic, analgesic, or anxiolytic effects, (d) Outcomes: prognostic outcomes including successful recanalization rate and neurological outcomes.

### Primary outcome, secondary outcomes, and definitions

#### Primary outcome

- Frequency of good functional outcome (i.e., functional independence), which was defined as one with a modified Rankin Scale (mRS) score of 0–2, at 3-month follow-up.

#### Secondary outcomes

- SRR following EVT. Successful recanalization referred to an achievement of an extended or modified thrombolysis in cerebral infarction (TICI) scale of 2b or 3.
- Risk of mortality within 3-months.
- Risk of symptomatic intracranial hemorrhage (ICH) during hospitalization.
- Risk of procedure-related complications.
- Risk of hypotension, the definition of which was according to that defined in individual studies.
- Risk of pneumonia.
- Neurological outcome at post-procedure 24–48 h assessed with the NIHSS.
- Time from puncture to successful recanalization, which referred to the period from groin puncture to arterial reperfusion.

### Analysis and assessment of risk of bias

Cochrane Review Manager (RevMan 5.3; Copenhagen: The Nordic Cochrane Center, The Cochrane Collaboration, 2014) was used for data synthesis. Risk ratios (RRs) or mean difference (MD) with 95% confidence intervals (CIs) were calculated based on a random effects model assuming heterogeneity across studies (20, 21). Heterogeneity was assessed with *I*^2^ statistics [i.e., low (*I*^2^ < 50%), moderate (*I*^2^ = 50–75%), and high (*I*^2^ > 75%)]. For studies with a high heterogeneity (*I*^2^ > 50%), a leave-one-out sensitivity analysis was conducted to evaluate stability of results (22). A probability value of <0.05 was considered statistically significant for all (including subgroup) analyses.

Two authors independently assessed the risk of bias for each study using the revised Cochrane risk-of-bias tool for randomized trials (RoB 2.0) (23) based on five domains, namely, possible bias from the randomization process, deviations from intended interventions, outcome measurement, missing outcome, and selection of the reported results.

To minimize false-positive results attributed to multiple testing and sparse data, trial sequential analysis (TSA) with TSA viewer version 0.9.5.10 Beta (www.ctu.dk/tsa) was conducted to test the robustness of the cumulative evidence as previously reported (24). Following the calculation of the required information size (RIS) and the trial sequential monitoring boundaries, the correlation between the cumulative Z curve and the TSA boundary was examined. To calculate the RIS for dichotomous outcomes, two-sided tests were adopted with a type I error, power, and a relative risk reduction being set at 5, 80, and 20%, respectively.

## Results

### Study selection and characteristics of included studies

**Figure 1:**
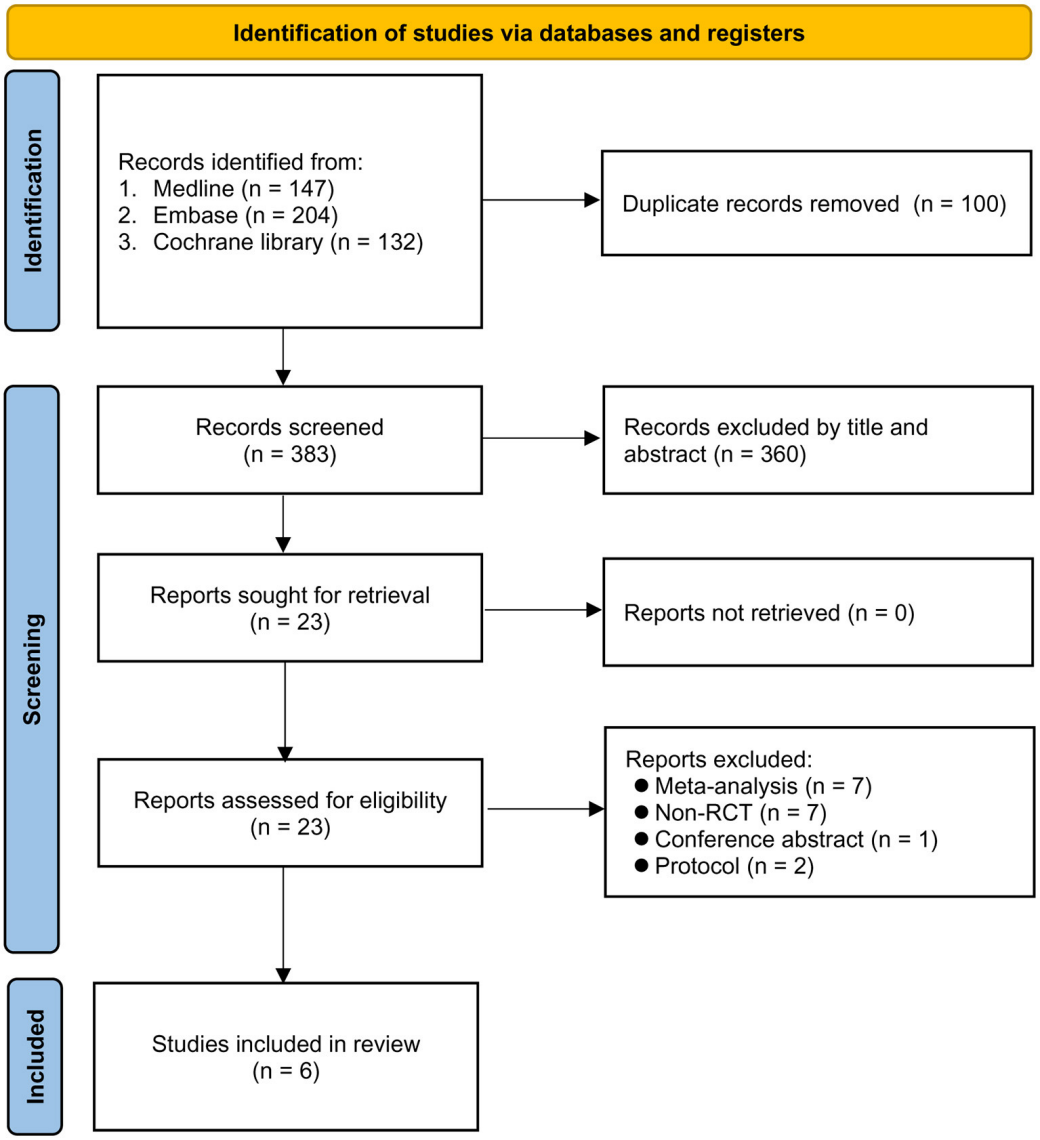
PRISMA flow diagram of study selection for the current meta-analysis.

**Figure 2:**
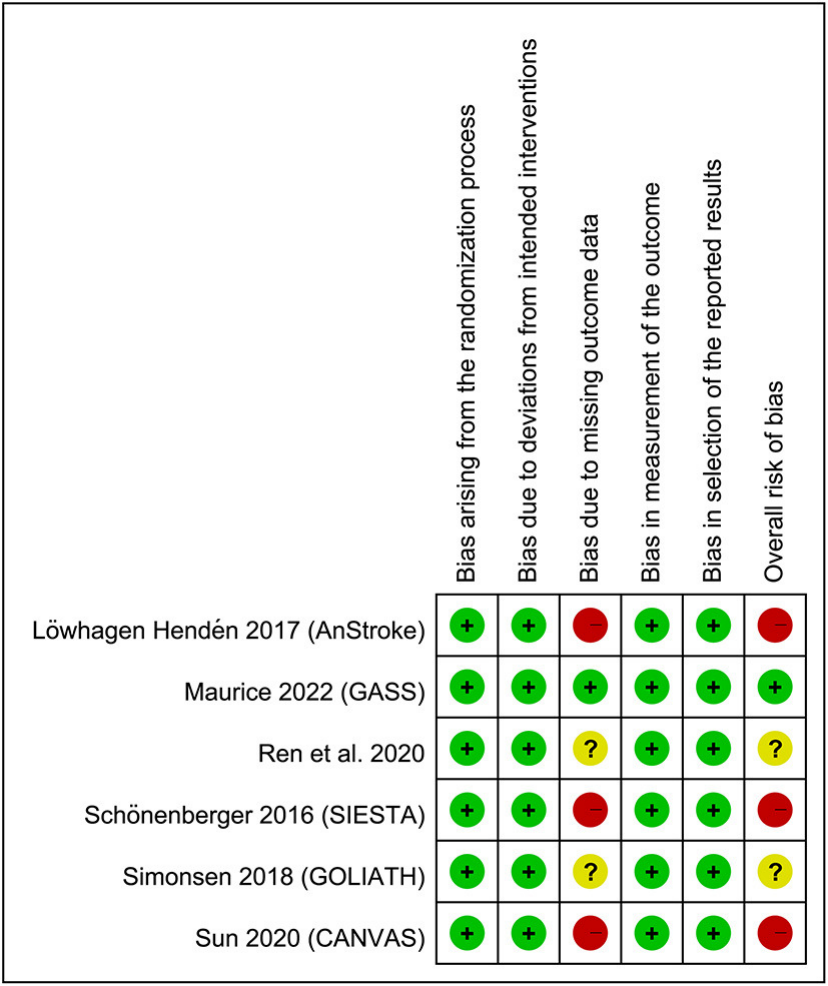
Summary of different categories of risk of bias of the included studies. Green: low risk of bias; yellow: moderate risk of bias; red: high risk of bias.

Figure 1 shows the various reasons for study exclusion after full-text screening. Finally, six RCTs involving 883 patients undergoing EVT published from 2016 to 2022 were included in this meta-analysis (14–19). Study characteristics are described in Table 1. The median or mean age of the participants ranged from 60 to 73 years with a proportion of male gender being 44–66%. The six studies that provided details regarding the baseline NIHSS score (range: 13–20) all reported no difference between the GA and CS groups (14–19). The sample size of individual RCT varied between 40 and 345. In the CS group, the anesthetic conversion rates were between 4.5 and 20% with a pooled incidence of 10.3% (Supplementary Figure 1). The reasons for conversion are demonstrated in Supplementary Table 2, revealing that patient agitation was the most common reason for conversion. In the GA group, anesthetic agents for maintenance of anesthesia included sevoflurane (one study) (15) and propofol (four studies) (16–19) with the use of remifentanil, while one study did not provide relevant details (14). In the CS group, propofol with or without short-acting opioids was used in the three studies (16–18). The two other trials only adopted short-acting opioids (i.e., remifentanil) to provide CS (15, 19), and one study did not report this information (14). Of the six studies included in the present meta-analysis, two provided information about brain infarct volume 3 days following acute stroke. One of the studies demonstrated a notable reduction in final infarct volume in the GA group compared to that in the CS group (22.3 vs. 38.0 mL, respectively, *p* = 0.04) despite a lack of significant difference in the initial infarct volume (10.5 vs. 13.3 mL) and infarct growth (8.2 vs. 19.4 mL) (16), while the other only showed comparable final infarct volume between the two groups (i.e., 20 vs. 20 mL) (15). The risks of bias of individual studies are summarized in Figure 2. The anesthetic conversion rate was 5–10% and ≥10% in two (16, 18) and three (14, 15, 17) studies, respectively. Accordingly, the risk of these studies were considered to be uncertain or high.

**Table 1:** Characteristics of studies (*n* = 6).

**Author-year (trial)**	**Age** **(years)**	**Male (%)**	**NIHSS** **Score at** **baseline**	**IV t-PA** **(%)**	**Endovascular technique**			**GA** **group**	**CS** **group**	**N**	**Conversion** **Rate** **(%)¶**	**Country**
					**A (%)**	**B (%)**	**C (%)**					
Löwhagen Hendén et al. (15) (Anstroke)	73 vs. 72	58 vs. 51	20 vs. 17	73 vs. 80	NR	NR	NR	S, R	R	90	15.6	Sweden
Maurice et al. (19) (GASS)	71 vs. 73	47 vs. 44	16 vs. 16	66 vs. 65	NR	NR	NR	P,R	R	345	4.5	France
Ren et al. (18)	69 vs. 69	54 vs. 57	14 vs. 14	77 vs. 81	NR	NR	NR	P, R, D	P,D,F	130	9.5	China
Schönenberger et al. (14) (SIESTA)	72 vs. 71	66 vs. 55	17 vs. 17	63 vs. 65	82 vs. 86	8 vs. 5	22 vs. 16	NR	NR	150	14.3	Germany
Simonsen et al. (16) (GOLIATH)	71 vs. 72	55 vs. 48	18 vs. 17	77 vs. 73	22 vs. 19	39 vs. 38	17 vs. 16	P,R	P,F	128	6.3	Demark
Sun et al. (17) (CANVAS)	67 vs. 60	65 vs. 65	14 vs. 13	45 vs. 55	10 vs. 15	40 vs. 45	50 vs. 40	P,R	P,Su	40	20	China

NR, not reported; GA, general anesthesia; CS, conscious sedation; IV, intravenous; NIHSS; A: stent retriever; B: direct aspiration; C: stent retriever combined with direct aspiration;

¶ Conversion to general anesthesia; NIHSS, National Institutes of Health Stroke Scale; P, propofol; F, fentanyl; R, remifentanil; D, dexmedetomidine; S, sevoflurane; Su, sufentanil.

### Outcomes

#### Primary outcomes

**Figure 3:**
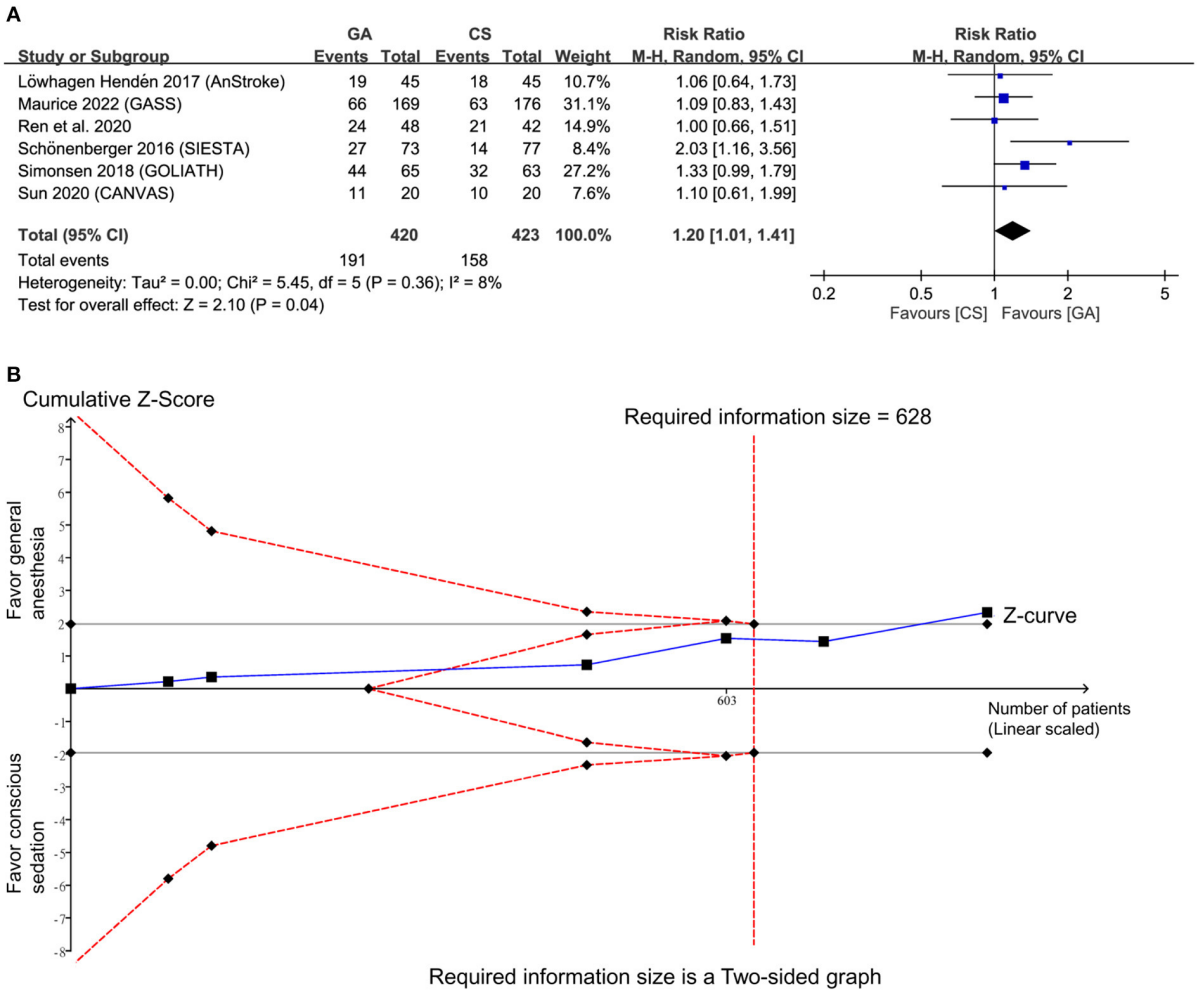
**(A)** Forest plot comparing the risk of good functional outcome between general anesthesia (GA) and conscious sedation (CS) groups. M-H, Mantel-Haenszel; CI, confidence interval; **(B)** Trial sequential analysis of risk of good functional outcome. The risk of type I error was set at 5% with a power of 80%.

Pooled analysis showed a high frequency of good functional outcomes (RR = 1.2, 95% CI: 1.01 to 1.41, *p* = 0.04; *I*^2^ = 8%) (Figure 3A) in the GA group compared to the CS group (14–19). Sensitivity analyses were not performed due to a low heterogeneity. Crossing of the cumulative Z-curve over RIS on TSA suggested sufficient evidence to reach a sound conclusion for this primary outcome (Figure 3B).

#### Secondary outcomes: Procedure-related outcomes

**Figure 4:**
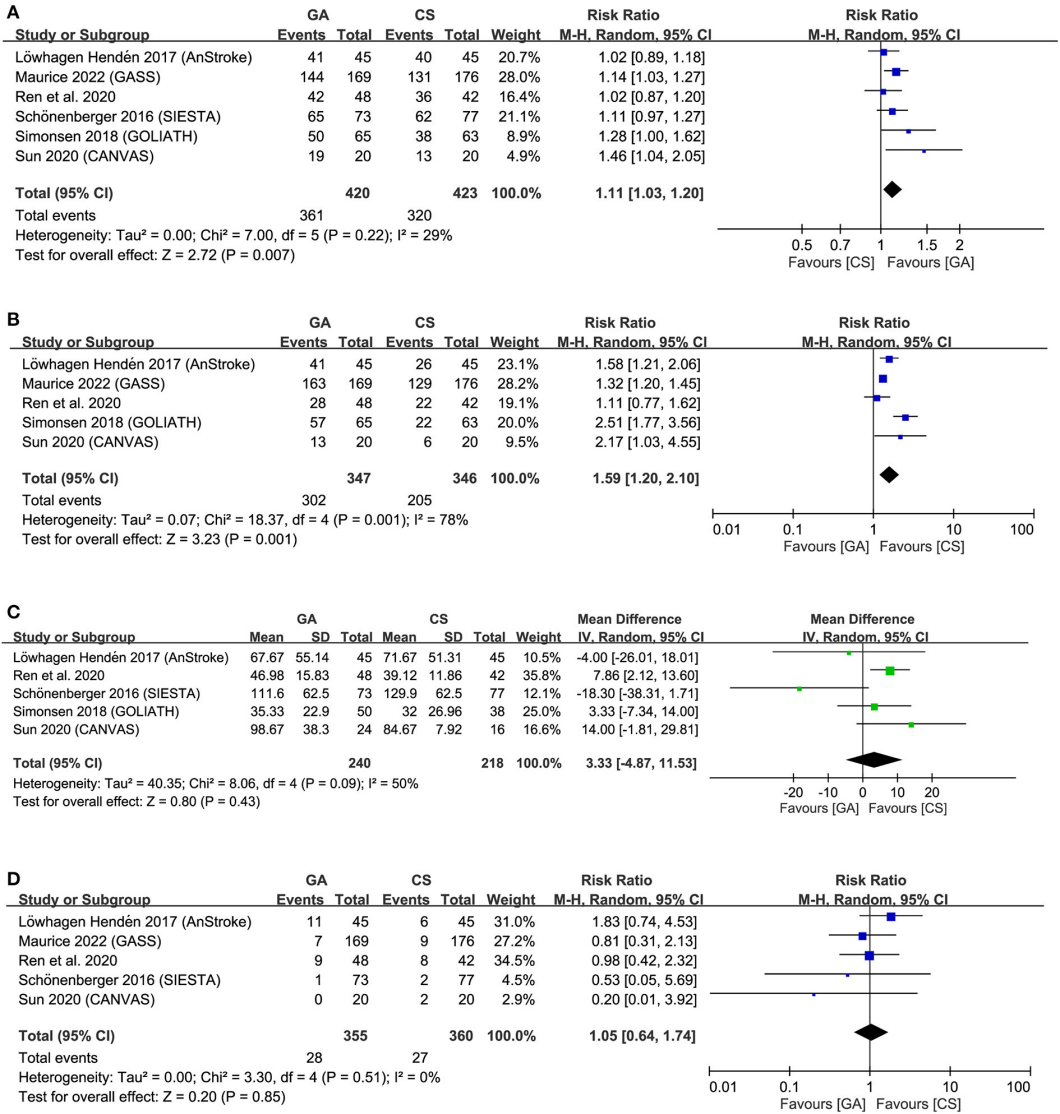
Forest plot comparing **(A)** successful recanalization rate, **(B)** hypotension risk, **(C)** duration from puncture to reperfusion, and **(D)** risk of procedure-related complications between general anesthesia (GA) and conscious sedation (CS) groups. IV, inverse variance; CI, confidence interval; M-H, Mantel-Haenszel.

Regarding procedural outcomes, the merged results revealed a higher SRR (RR = 1.11, 95% CI: 1.03 to 1.2, *p* = 0.007; *I*^2^ = 29%) in the GA group than that in the CS group (Figure 4A). However, patients receiving GA also had a higher hypotension risk compared to those undergoing CS for EVT (RR = 1.59, 95% CI: 1.2 to 2.1, *p* = 0.001, *I*^2^ = 78%) (Figure 4B). Sensitivity analysis for this outcome demonstrated a consistent finding when certain studies were removed one at a time. Nevertheless, the cumulative duration of hypotension episode was comparable between the two groups (MD = 0.86 min, 95% CI: −1.78 to 3.5, *p* = 0.52, *I*^2^ = 0) (Figure not shown). No significant difference was noted in the duration from puncture to reperfusion (MD = 3.33, 95% CI: −4.87 to 11.53, *p* = 0.43, *I*^2^ = 50%) (Figure 4C) and the risk of procedure-related complications (RR = 1.05, 95% CI: 0.64 to 1.74, *p* = 0.85, *I*^2^ = 0%) between the two groups (Figure 4D). Sensitivity analyses were not performed for other outcomes because of a low heterogeneity.

Crossing of the cumulative Z-curve over the trial sequential monitoring boundary in two outcomes (i.e., SRR, and risk of hypotension) (Supplementary Figures 2, 3) on TSA indicated sufficient evidence for these three outcomes to reach a firm conclusion. In contrast, TSA for puncture to reperfusion time and procedure-related complications demonstrated a failure of interaction between the cumulative Z-curve and the futility boundary (Supplementary Figures 4, 5), implicating insufficient evidence for a robust conclusion.

#### Secondary outcomes: Other prognostic outcomes

**Figure 5:**
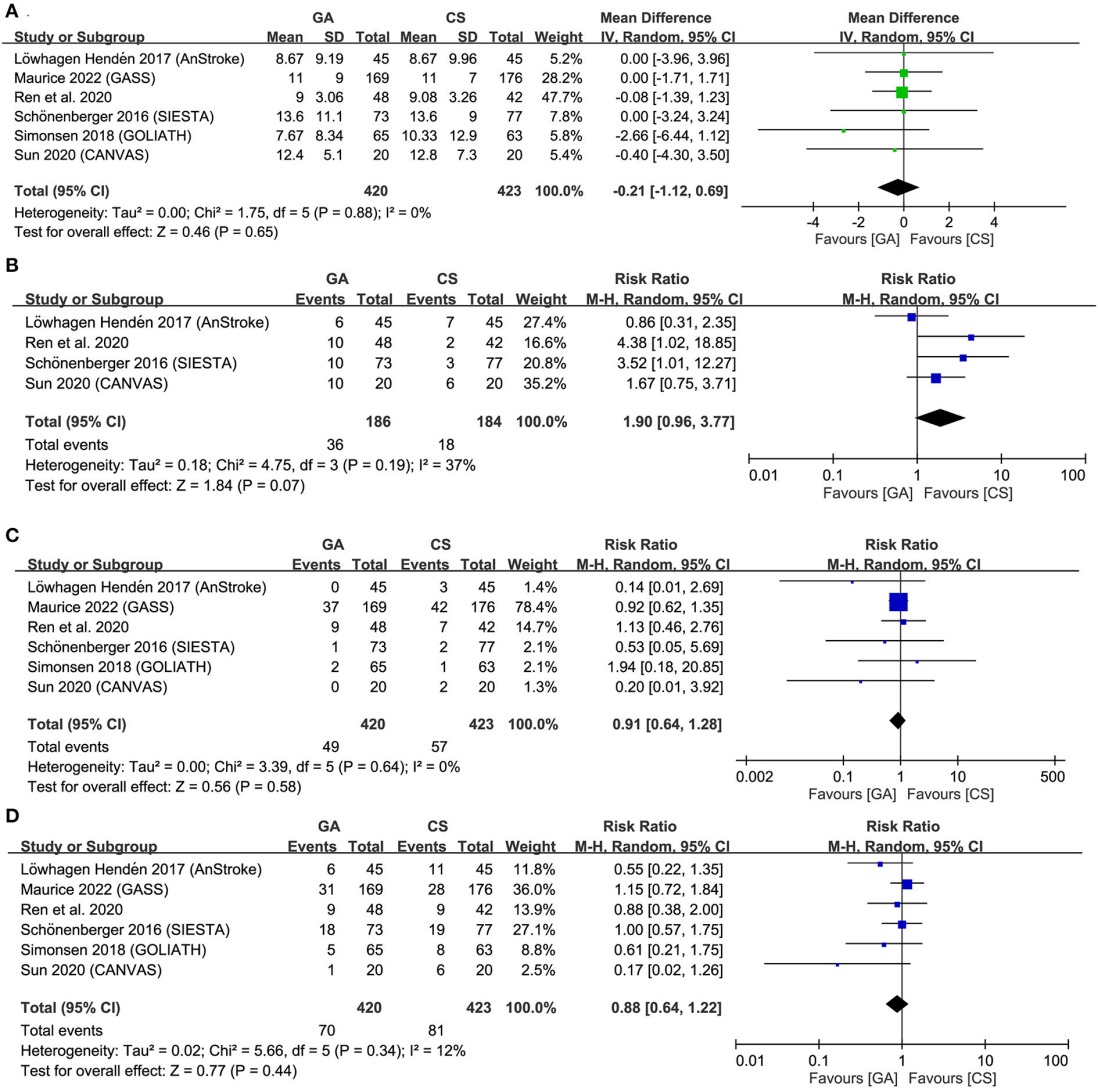
Forest plot comparing **(A)** National Institutes of Health Stroke Scale (NIHSS) score at 24–48 h, **(B)** risk of pneumonia, **(C)** symptomatic intracranial hemorrhage (ICH), and **(D)** mortality at 3-month follow-up between general anesthesia (GA) and conscious sedation (CS) groups. IV, inverse variance; CI, confidence interval.

Forest plots demonstrated no significant difference in NIHSS score at 24–48 h (MD = −0.21, 95% CI: −1.12 to 0.69, *p* = 0.65, *I*^2^ = 0) (Figure 5A) as well as the risks of pneumonia (RR = 1.9, 95% CI: 0.96 to 3.77, *p* = 0.07, *I*^2^ = 37%) (Figure 5B), symptomatic ICH (RR = 0.91, 95% CI: 0.64 to 1.28, *p* = 0.58, *I*^2^ = 0) (Figure 5C), and mortality at 3-month follow-up (RR = 0.88, 95% CI 0.64 to 1.22, *p* = 0.44; *I*^2^ = 12%) (Figure 5D) between the two groups. Sensitivity analyses were not performed due to a low heterogeneity for all the outcomes. TSA for difference in NIHSS score was ignored because of inadequate information for TSA boundary construction (Supplementary Figure 6). For the risks of pneumonia, symptomatic ICH, and mortality rate, failure of the cumulative Z-curve to cross the trial sequential monitoring boundary or the futility boundary suggested inconclusive evidence for these outcomes (Supplementary Figures 7–9).

## Discussion

Focusing on patients with AIS undergoing EVT, our results revealed a higher successful recanalization rate in GA compared with CS groups (85.7% vs. 75.7%, respectively) with similar duration of puncture to reperfusion and risk of procedure-related complications. There was also no difference in the immediate neurological outcome (i.e., NIHSS score at 24–48 h) between the GA and CS groups. Nevertheless, the 3-month neurological prognosis (i.e., functional independence) was better in the former than the latter (45.5% vs. 37.4%, respectively) without significant differences in the risks of 3-month mortality, symptomatic ICH, and pneumonia between the two groups. Despite a higher incidence of hypotension episodes with the use of GA, there was no difference in the accumulative period of hypotension between the two groups. The pooled conversion rate from CS to GA was 10.3%.

Our updated meta-analysis including six RCTs demonstrated that the use of GA was associated with a higher recanalization rate and more favorable functional outcome compared to the use of CS during EVT. The lack of a significant difference in baseline NIHSS score between the two groups together with our findings of better outcomes in the GA group compared to the CS group further supported the superiority of GA to CS in this clinical setting. Indeed, the worse treatment outcome among patients with acute stroke who underwent GA than in those receiving CS in early studies has been found to be attributable to selection bias as those with a more severe condition tended to receive GA for EVA (25). Although our main findings were consistent with those of a previous meta-analysis (7), the advantages of the present investigation included the enrollment of more participants (i.e., 883 patients) and the use of TSA to examine the robustness of our evidence. Our findings on functional outcome were inconsistent with those of a recent meta-analysis that included seven RCTs (anterior cranial circulation, *n* = 6; posterior cranial circulation, *n* = 1) for comparing the impact between GA and non-GA in patients with AIS receiving EVT. While the current study showed a significant association between GA with 3-month functional outcome compared with CS, the recent meta-analysis demonstrated no difference between the GA and non-GA groups (i.e., conscious sedation, local anesthesia, monitored anesthesia care) (26). The discrepancy in results may be explained by the differences in the number of RCTs included for functional outcome analysis; while the current study extracted relevant information from six RCTs (14–19), the recent meta-analysis only focused on four of our six included studies (14, 15, 17, 19). Further examination of the adequacy of patient sample size of the previous meta-analysis (26) with TSA indicated no crossing between the z-curve and the RIS (data not shown), suggesting insufficient evidence to reach a sound conclusion for this outcome (26). In contrast, TSA of the current study demonstrated a satisfactory sample size for reaching a robust conclusion (Figure 3).

Several factors may contribute to the favorable neurological outcome (i.e., functional independence at 3-months) in the GA group in the current study. Although a number of studies have reported an association between 3-month neurological outcome and recanalization rate (2, 7, 8, 27), the higher recanalization rate in the GA group may only be one of the possible explanations for our promising outcome. In fact, some authors suggested that the possible neuroprotective effects of anesthetic agents being used in GA may be more important contributors to a better outcome compared to a high recanalization rate (28). Consistently, a previous meta-analysis demonstrated that the use of GA was associated with a better neurological outcome compared to those with CS for patients with recanalization failure, supporting the potentially neuroprotective effect of GA (28). Such a neuroprotective action of GA against ischemic brain infarct was further underscored by a reduced final infarct volume and infarct growth in one of our included trials despite the lack of statistical significance of the latter (16). In concert with this proposal, previous clinical and animal studies have reported a neuroprotective effect of anesthetic agents (29). For instance, propofol has been found to be protective against ischemia-reperfusion injury through suppressing oxidative stress-related astrocyte injuries, reinforcing astroglial-mediated neuronal defense (29), reducing cerebral metabolism, enhancing antioxidant ability, and redirecting cerebral blood flow to focal ischemic penumbra area (30). Nevertheless, one of the novel findings of the current meta-analysis was a higher recanalization rate in the GA group compared to that in the CS group without a significant difference in immediate post-procedural NIHSS between the two groups. Although the subsequent more significant improvement in neurological outcome at 3-months in the GA group than that in the CS group may still support a long-term beneficial influence of anesthetics as propofol was used for anesthetic maintenance in four out of our six included studies (16–19), the higher EVT recanalization rate in the former may contribute to the favorable outcome.

Despite potential benefit from propofol, concurrent use of opioid with propofol may lead to respiratory depression and subsequent hypercapnia in the CS group in the current meta-analysis. A hypocapnic state has been shown to widen the plateau region of the autoregulatory curve (31), thereby improving cerebrovascular autoregulatory capacity to maintain a constant CBF in the face of fluctuations in cerebral perfusion pressure (32). Improving the autoregulatory capacity of cerebrovascular is of particular importance in disease situations such as acute stroke in which the patient may experience extremes of cerebral perfusion pressure from rising intracranial pressures or uncontrolled hypertension (32). In this way, our finding of a poorer 3-month neurological outcome in the CS group compared to patients undergoing GA for EVT may support this argument, taking into consideration the possibility of respiratory depression-induced hypercapnia in patients receiving CS.

Several retrospective studies reported that hypotension during the procedure is a poor prognostic factor for EVT (3, 4, 33). In the present meta-analysis, although the risk of hypotension was higher in the GA compared to the CS groups, there was no difference in the cumulative duration of hypotension attack. Our apparently contradictory finding of more significantly improved functional outcome in the GA group compared to patients subjected to CS may suggest a relatively minor role of hypotension provided that there was no prolonged hypotensive episode as well as related complications. Accordingly, our results implied that the beneficial effect of GA may outweigh its associated risk of hypotension given that the patients are monitored under strict protocols. Therefore, in patients scheduled for EVT under GA, a well-designed management strategy for hemodynamic instability should be incorporated into the peri-procedural care protocol to optimize neurological outcome.

Despite a substantially lower anesthetic conversion rate compared with patients receiving local anesthesia without sedation (i.e., 17.5%) (34), the conversion rate in the current meta-analysis remained high at ~10.3%, which was comparable to that reported in a previous meta-analysis of retrospective studies (i.e., 8.8%) (34). Conversion from a non-GA approach to GA is known to prolong the procedural time and have a theoretical detrimental effect on neurological outcomes as described in a retrospective clinical report on the effect of conversion from CS to GA (9). Taking into account the high conversion rate from a non-GA approach, GA may be the first choice for patients who are scheduled for EVT to minimize the risk of procedural delay. Nevertheless, despite our finding of a tendency of an increased pneumonia risk in patients receiving GA compared to CS based on a random-effects model, it failed to reach statistical significance. Our result was inconsistent with that of a previous meta-analysis that used a fix-effects model and demonstrated a significant increase in risk of pneumonia among patients receiving GA for EVT (7). Because TSA in the current meta-analysis suggested inconclusive evidence, this finding remains a concern for patients receiving GA for EVT.

There are some limitations in our study. First, the sample size of only six RCTs was not large enough to reach a sound conclusion. Besides, most were single-center studies with well-trained neuro-anesthetic teams which may not be available in a real world scenario. Second, because the depth of sedation varies with the goals of CS, the lack of a standardized sedation goal for EVT may affect the conversion rate from CS to GA which, in turn, could influence the risk of poor clinical outcome (9). Third, the use of different anesthetic agents may bias our results. For instance, there were four RCTs using propofol and one choosing sevoflurane for anesthetic maintenance, while the other did not give details regarding anesthetic agents (14–19). Volatile agents such as sevoflurane have a vasodilatory effect which may worsen clinical outcome by diverting intracranial blood flow away from the ischemic penumbra area, especially in the presence of systemic hypotension (30). Nevertheless, the low heterogeneity across our studies suggested robustness of our findings. Fourth, a previous meta-analysis reported that although the choice of thrombectomy technique (i.e., direct aspiration approach vs. stent-retriever) had no influence on the rate of successful recanalization, a better functional outcome at 3-months was noted in patients receiving direct aspiration (35). In the current meta-analysis, the proportion of patients receiving direct aspiration ranged from 8 to 40% in three trials, while the other three studies did not provide relevant details. Therefore, potential confounding effects from the use of different thrombectomy techniques across our included studies cannot be ruled out. Finally, because we did not include one ongoing trial (Sedation vs. General Anesthesia for Endovascular Therapy in Acute Ischemic Stroke; SEGA, NCT 03263117) in the present meta-analysis because of the unavailability of data for analysis, the impact of the outcomes of that study on the pooled results remains to be elucidated.

## Conclusion

Among patients with acute ischemic stroke from large vessel occlusion in the anterior circulation, endovascular thrombectomy under general anesthesia was associated with a higher successful recanalization rate as well as a better 3-month neurological outcome compared to the use of conscious sedation. Because of the small effect size and the tendency toward an increased pneumonia risk related to general anesthesia, whether the benefit of general anesthesia outweighs its risk remains to be elucidated.

## Data availability statement

The original contributions presented in the study are included in the article/Supplementary material, further inquiries can be directed to the corresponding author/s.

## Author contributions

C-WL and K-CH: conceptualization, literature search, and data extraction. Y-TH and S-ZW: methodology. Y-PC and Y-TH: trial selection. C-HH and M-HC: data analysis. K-CH, C-WL, and C-KS: writing—original draft preparation. K-CH and C-KS: writing—review and editing. All authors have read and agreed to the published version of the manuscript. All authors contributed to the article and approved the submitted version.

## Conflict of interest

The authors declare that the research was conducted in the absence of any commercial or financial relationships that could be construed as a potential conflict of interest.

## Publisher's note

All claims expressed in this article are solely those of the authors and do not necessarily represent those of their affiliated organizations, or those of the publisher, the editors and the reviewers. Any product that may be evaluated in this article, or claim that may be made by its manufacturer, is not guaranteed or endorsed by the publisher.

## Abbreviations

EVT: endovascular thrombectomy
AIS: acute ischaemic stroke
CS: conscious sedation
GA: general anesthesia
mRS: modified Rankin Scale
TICI: thrombolysis in cerebral infarction
NIHSS: National Institutes of Health Stroke Scale
RR: risk ratio
MD: mean difference
RCT: randomized controlled study
SRR: successful recanalization rate
ICH: intracranial hemorrhage.
