# Supplementary material for: General anesthesia but not conscious sedation improves functional outcome in patients receiving endovascular thrombectomy for acute ischemic stroke: A meta-analysis of randomized clinical trials and trial sequence analysis
Source: Front Neurol. 2022 Sep 14;13:1017098. doi: 10.3389/fneur.2022.1017098 (PMC9515609; doi:10.3389/fneur.2022.1017098)

**Supplemental materials**

**Supplemental Table 1.** Search strategy for Medline

| Database | # | Search syntax |
| --- | --- | --- |
| Medline | 1 | ("endovascular thrombectomy" or "mechanical thrombectomy" or "Ischemic Stroke" or "stroke" or "Thrombectomy" or "Endovascular" or "Mechanical Thrombolysis").mp. |
|  | 2 | exp "Ischemic Stroke"/ or exp "Brain Ischemia"/ or exp "Thrombectomy"/ or exp "Endovascular Procedures"/ |
|  | 3 | General anesthesia.mp. |
|  | 4 | exp "Anesthesia, General"/ |
|  | 5 | ("Conscious sedation" or "Sedation").mp. |
|  | 6 | exp "Conscious Sedation"/ |
|  | 7 | (1 or 2) and (3 or 4) and (5 or 6) |
|  | 8 | 7 and (((randomized controlled trial or controlled clinical trial).pt. or randomi*ed.ab. or placebo.ab. or drug therapy.fs. or randomly.ab. or trial.ab. or groups.ab.) not (exp animals/ not humans.sh.)) |

**Supplemental Table 2.** Reasons for conversion to general anesthesia

| Study/year | Trial | Reasons |
| --- | --- | --- |
| Löwhagen Hendén 2017 | Anstroke | NR |
| Maurice 2022 | GASS | Conscious sedation was converted into general anesthesia for eight patients (4%) for the following reasons: agitation (n = 4); vomiting (n = 1); Glasgow coma scale less than 8 (n = 1); hypoxemia (n = 1); or other (failure of catheter, respiratory arrest; n = 3). |
| Ren 2020 |  | NR |
| Schönenberger 2016 | SIESTA | Severe agitation (n=7)  Apnea after sedation bolus (n=2)  Respiratory insufficiency (n=1)  Direct puncture of internal carotid artery (n=1) |
| Simonsen 2018 | GOLIATH | Four patients (6.3%) in the CS group converted to the GA group due to movement. Two of these patients also vomited, and 1 experienced desaturation due to aspiration. |
| Sun 2020 | CANVAS | Four patients (18.2%) in the CS group had to be converted to GA after randomization because of significant agitation. |

NR: not report

**Supplemental Figure 1.** Conversion rate for the patients receiving conscious sedation


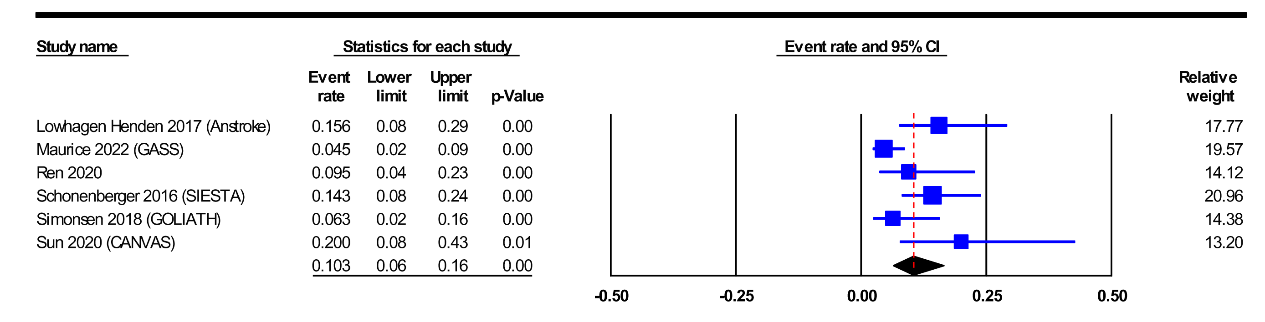


**Supplemental Figure 2.** Trial sequential analysis for successful revascularization rate between both groups.


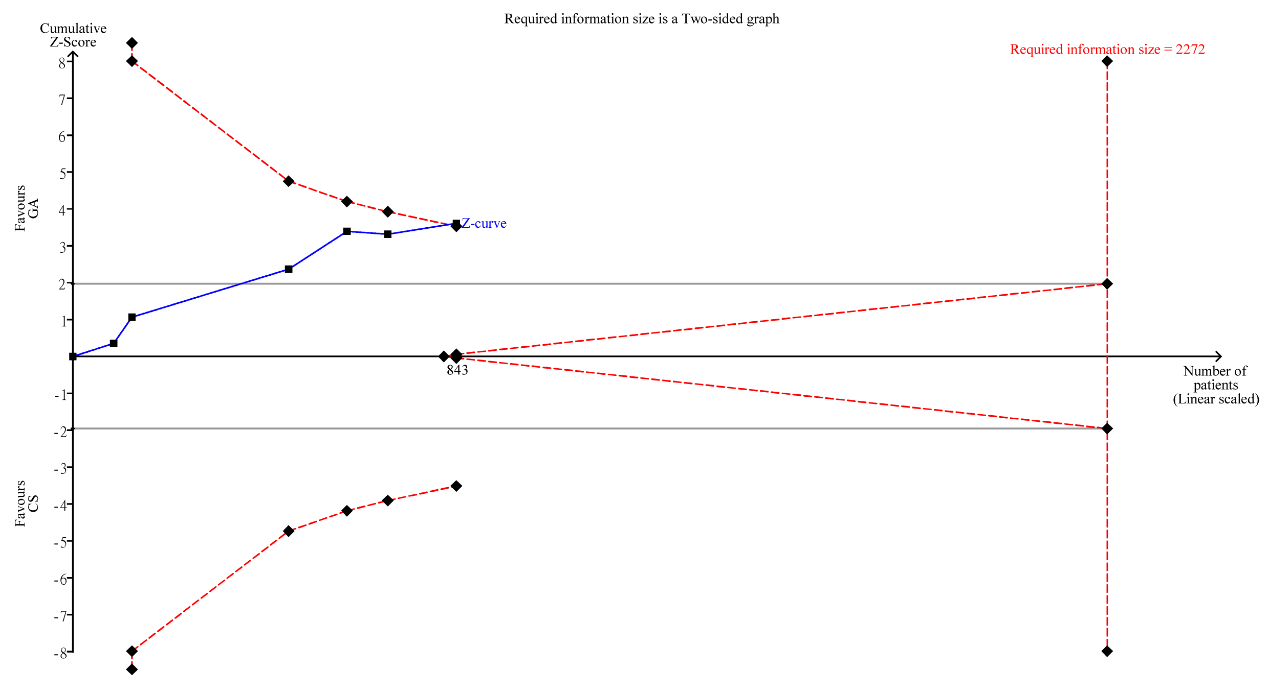


**Supplemental Figure 3.** Trial sequential analysis for risk of hypotension between both groups.


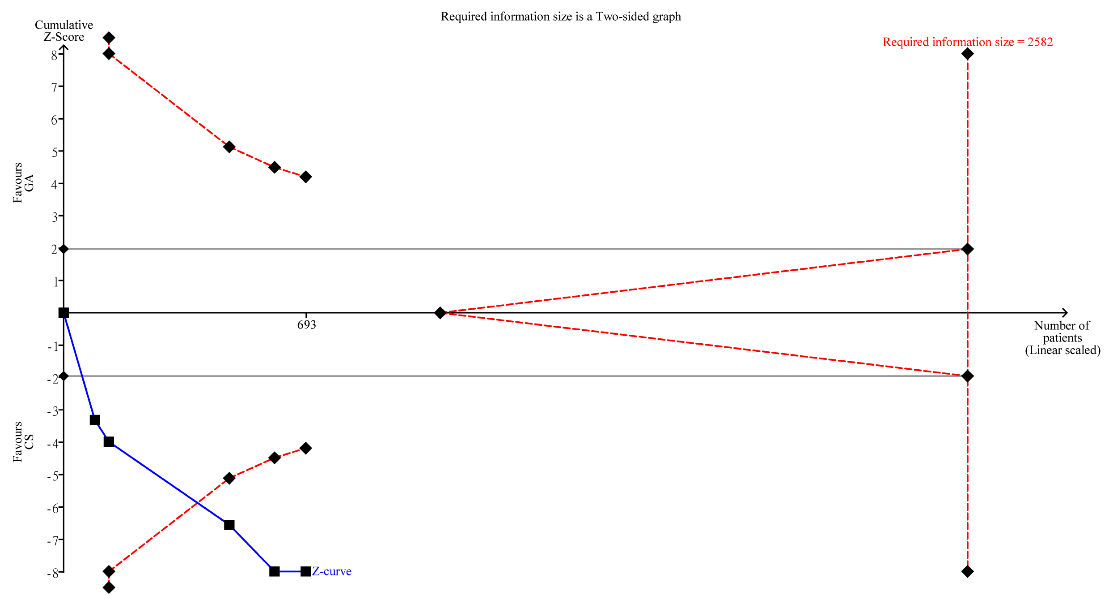


**Supplemental Figure 4.** Trial sequential analysis for puncture to reperfusion time between both groups.


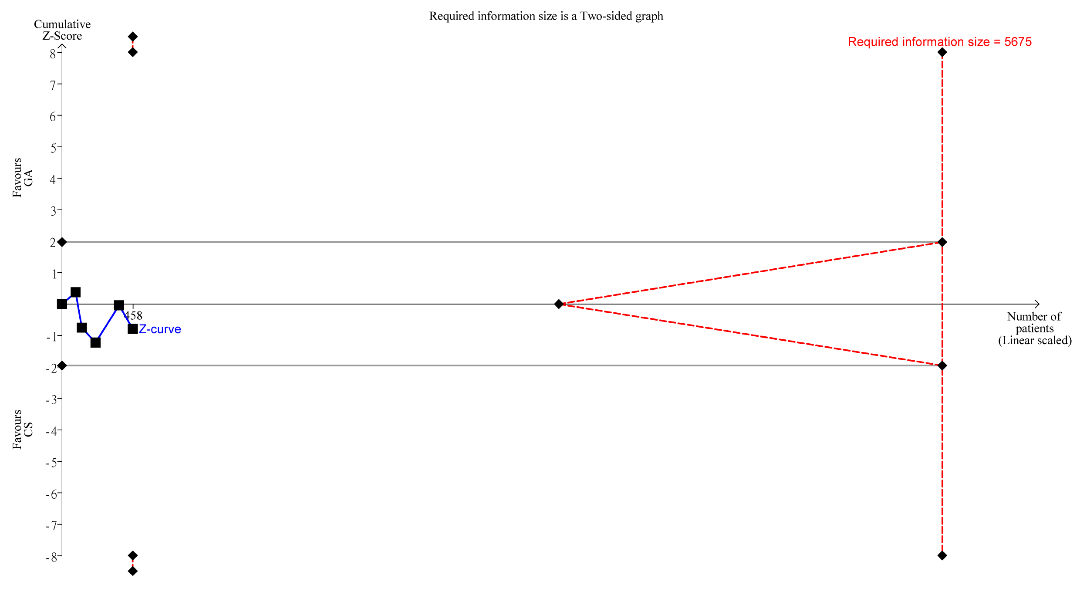


**Supplemental Figure 5.** Trial sequential analysis for risk of procedure-related complications between both groups.


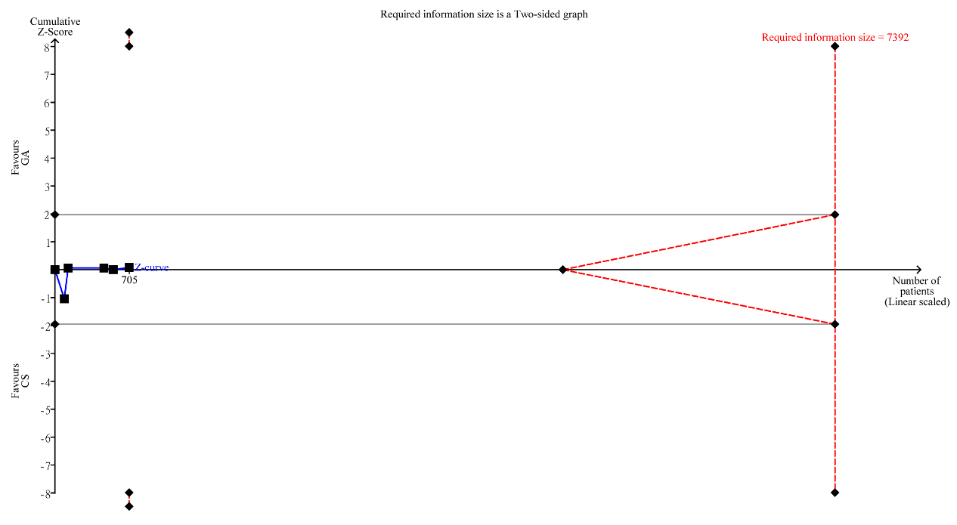


**Supplemental Figure 6.** Trial sequential analysis for NIHSS scores between both groups.


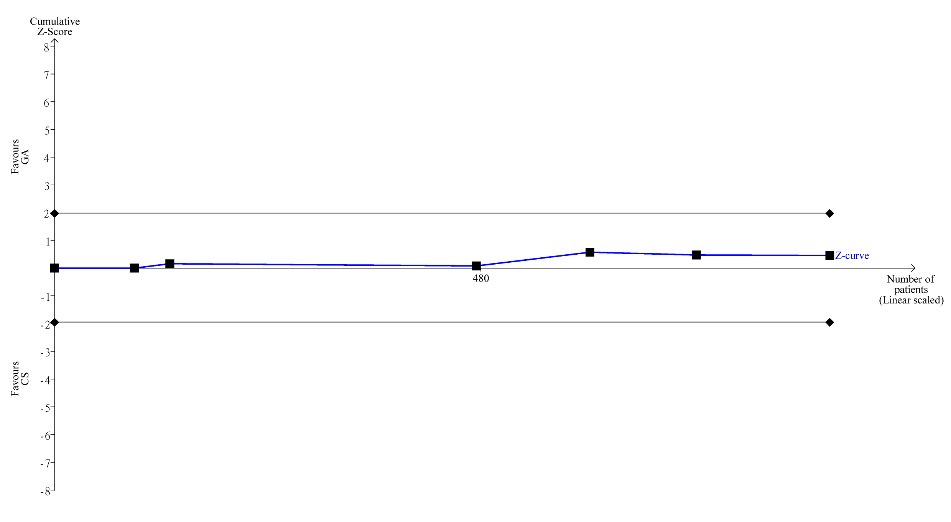


**Supplemental Figure 7.** Trial sequential analysis for risk of pneumonia between both groups.


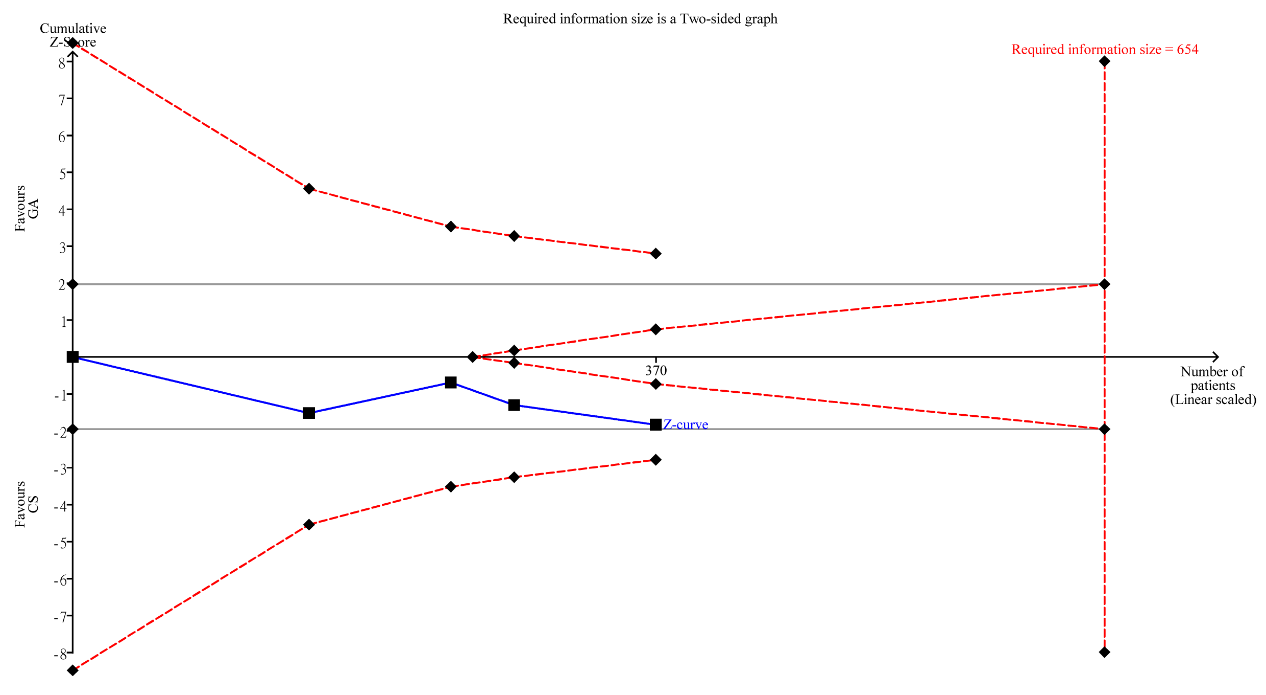


**Supplemental Figure 8.** Trial sequential analysis for risk of symptomatic intracranial hemorrhage between both groups.


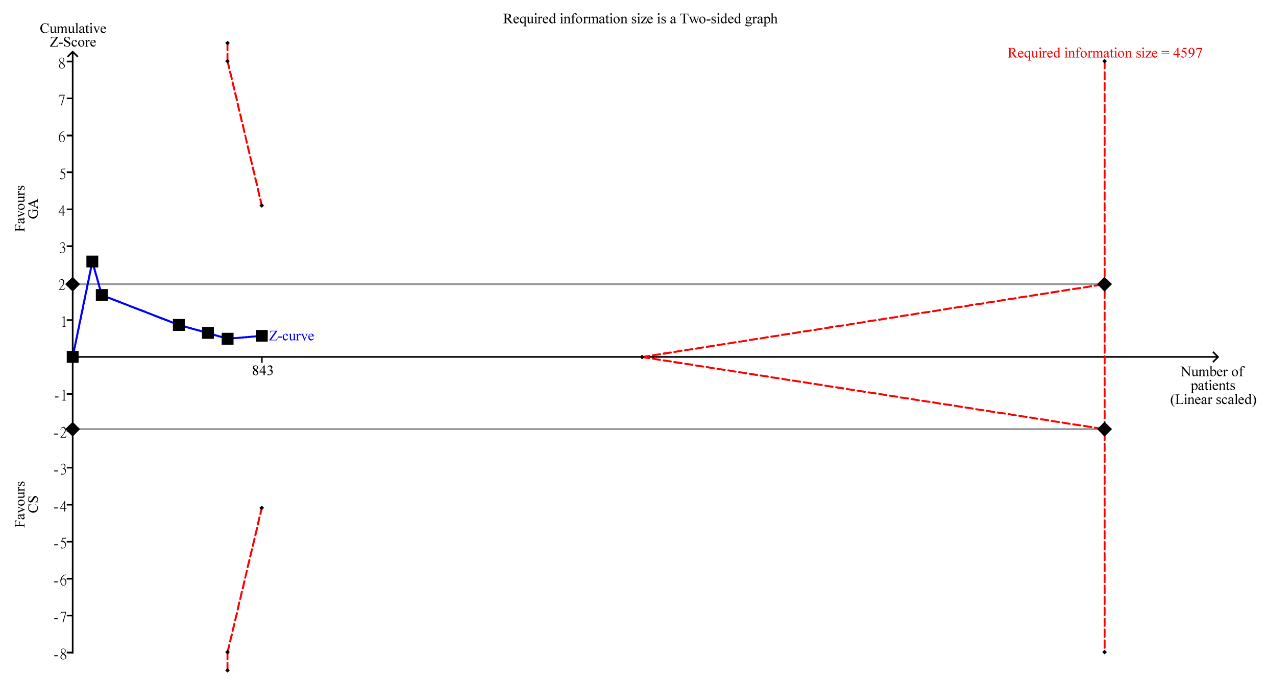


**Supplemental Figure 9.** Trial sequential analysis for risk of mortality at 3 months between both groups.


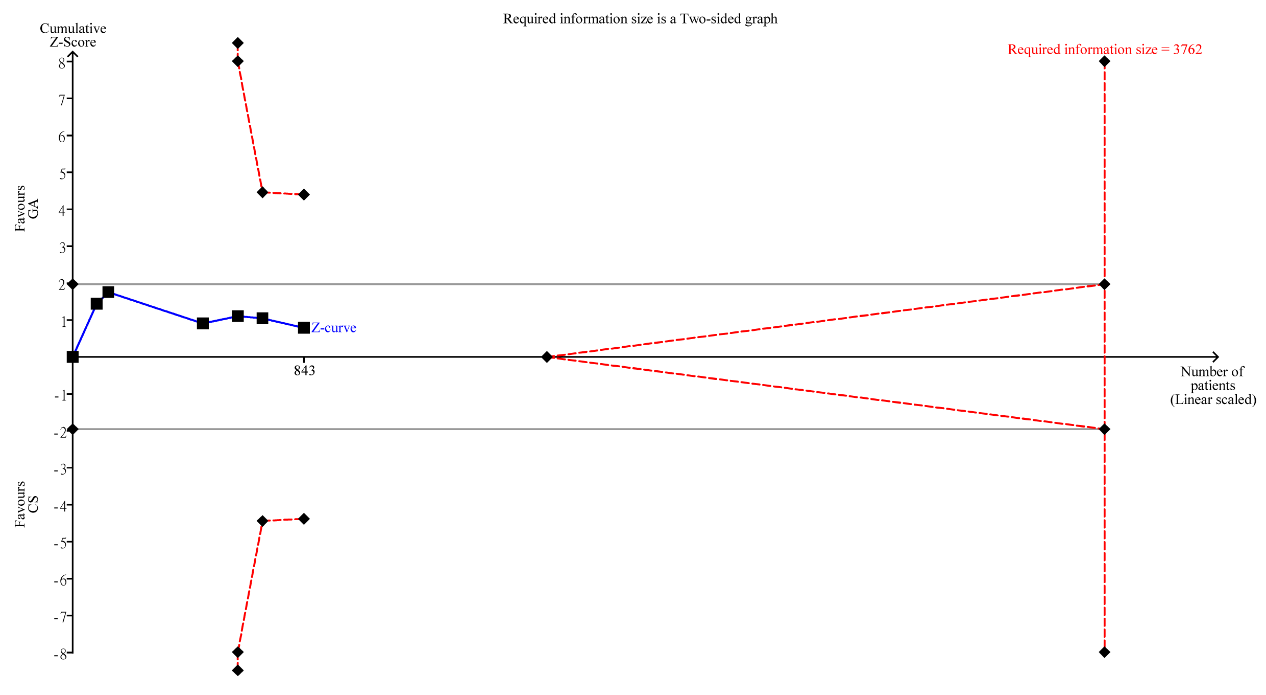

Supplement: Supplementary file 1 [file Data_Sheet_1.docx]
